# The synthetic neuroactive steroid SGE-516 reduces seizure burden and improves survival in a Dravet syndrome mouse model

**DOI:** 10.1038/s41598-017-15609-w

**Published:** 2017-11-10

**Authors:** Nicole A. Hawkins, Michael Lewis, Rebecca S. Hammond, James J. Doherty, Jennifer A. Kearney

**Affiliations:** 1 Department of Pharmacology Northwestern University Feinberg School of Medicine, Chicago, IL USA; 2 Drug Discovery and Development Sage Therapeutics, Cambridge, MA USA

## Abstract

Dravet syndrome is an infant-onset epileptic encephalopathy with multiple seizure types that are often refractory to conventional therapies. Treatment with standard benzodiazepines like clobazam, in combination with valproate and stiripentol, provides only modest seizure control. While benzodiazepines are a first-line therapy for Dravet syndrome, they are limited by their ability to only modulate synaptic receptors. Unlike benzodiazepines, neuroactive steroids potentiate a wider-range of GABA_A_ receptors. The synthetic neuroactive steroid SGE-516 is a potent positive allosteric modulator of both synaptic and extrasynaptic GABA_A_ receptors. Prior work demonstrated anticonvulsant activity of SGE-516 in acute seizure assays in rodents. In this study, we evaluated activity of SGE-516 on epilepsy phenotypes in the *Scn1a* ^+/−^ mouse model that recapitulates many features of Dravet syndrome, including spontaneous seizures, premature death and seizures triggered by hyperthermia. To evaluate SGE-516 in *Scn1a* ^+/−^ mice, we determined the effect of treatment on hyperthermia-induced seizures, spontaneous seizure frequency and survival. SGE-516 treatment protected against hyperthermia-induced seizures, reduced spontaneous seizure frequency and prolonged survival in the *Scn1a* ^+/−^ mice. This provides the first evidence of SGE-516 activity in a mouse model of Dravet syndrome, and supports further investigation of neuroactive steroids as potential anticonvulsant compounds for refractory epilepsies.

## Introduction

Dravet syndrome is a severe infant-onset epileptic encephalopathy most often caused by *de novo* mutation of *SCN1A* resulting in heterozygous loss-of-function of the Nav1.1 voltage-gated sodium channel^1^. Patients experience multiple seizure types in addition to delays of psychomotor and cognitive development^2^. Treatment with standard benzodiazepines like clobazam or clonazepam, in combination with additional anticonvulsants, has shown some success in managing chronic seizures^3^. While benzodiazepines are utilized as a first line therapy in Dravet syndrome, they are only capable of binding to GABA_A_ receptors at the α and γ subunit interface, limiting their ability to modulate both synaptic and extrasynaptic receptors^4^. Unlike benzodiazepines, neuroactive steroids have been shown to modulate both synaptic and extrasynaptic GABA_A_ receptors, including those containing δ or lacking γ subunits, and benzodiazepine-insensitive α subunits^5,6^. The neurosteroids alphaxalone and allopregnanolone have demonstrated protective effects in animal models against seizures or status epilepticus induced by pentylenetetrazol (PTZ), kaniate, bicuculline, pilocarpine and in the 6 Hz psychomotor test^7–10^. A new generation of synthetic neuroactive steroids was generated to improve the pharmacokinetic profile of endogenous neurosteroids^11^. One compound, SGE-516, showed increased oral bioavailability while still maintaining high potency as a GABA_A_ positive allosteric modulator (PAM)^11^. Prior work in animal models demonstrated the ability of SGE-516 to protect against acute seizures induced by PTZ and the 6 Hz psychomotor seizure model^12^. Furthermore, SGE-516 was shown to be anticonvulsant in the mouse corneal kindling model and the organophosphorus nerve agent model of status epilepticus^12,13^.

In this study, we evaluated the ability of SGE-516 to improve epilepsy phenotypes in the *Scn1a* ^+/−^ mouse model of Dravet syndrome. *Scn1a* ^+/−^ mice recapitulate many features of Dravet syndrome, including spontaneous seizures, premature death and seizures induced by hyperthermia. Using a series of optimized phenotyping assays, we recently demonstrated that the response profile of *Scn1a* ^+/−^ mice to standard anticonvulsant therapies is similar to reported drug responses of children with Dravet syndrome^14^. Here we evaluated SGE-516 in the *Scn1a* ^+/−^ Dravet mouse model and studied the effect of SGE-516 treatment on hyperthermia-induced seizures, spontaneous generalized tonic-clonic seizure (GTCS) frequency and survival. We found that treatment of *Scn1a* ^+/−^ mice with SGE-516 resulted in significant phenotype improvement, including reduced seizure burden and improved survival.

## Results

### Evaluation of SGE-516 against thermally-induced seizures

**Figure 1:**
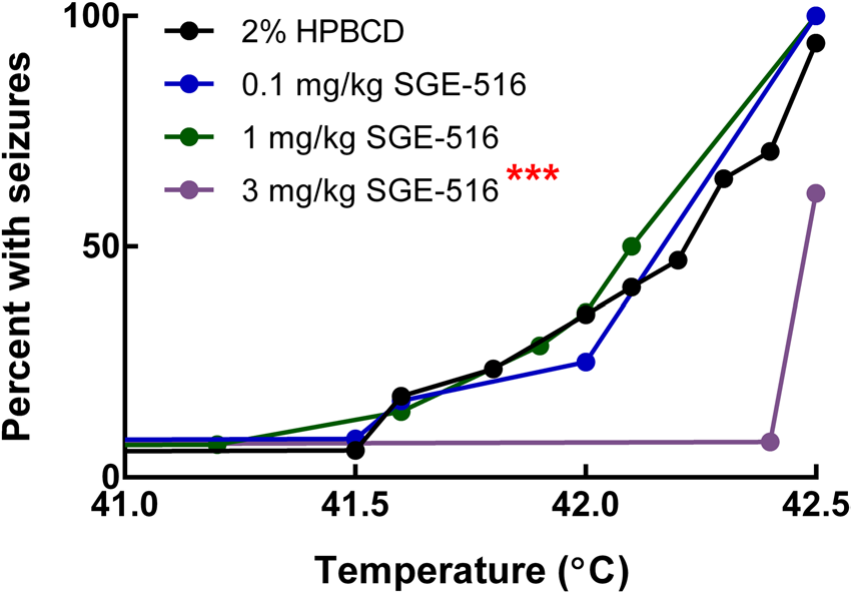
Evaluation of SGE-516 against hyperthermia-induced seizures. Cumulative seizure incidence curve for *Scn1a* ^+/−^ mice following acute administration of SGE-516 and hyperthermia-induced seizure threshold testing. An acute dose of 3 mg/kg SGE-516 significantly shifted the temperature threshold curve for hyperthermia-induced seizures (***p < 0.0005) P-values were determined by LogRank Mantel-Cox with *n* = 12–17 per treatment group.

Similar to individuals with Dravet syndrome, seizures can be provoked in *Scn1a* ^+/−^ mice by elevated body temperature. To determine the effect of SGE-516 on hyperthermia-induced seizures in *Scn1a* ^+/−^ Dravet mice, SGE-516 or vehicle (2% 2-Hydroxypropyl-β-cyclodextrin) was acutely administered 45 minutes prior to the initiation of a hyperthermia paradigm in which core body temperature was slowly elevated until onset of the first clonic convulsion with loss of posture or until 42.5 °C was maintained for 5 minutes. Mice that did not exhibit a GTCS during the protocol were considered seizure-free. SGE-516 was administered by intraperitoneal (IP) injection at doses of 0.1, 1 and 3 mg/kg based on the previously reported data from acute induced-seizure models^12^. Pre-treatment with 0.1 or 1 mg/kg SGE-516 did not have a significant effect on hyperthermia-induced seizures, while the 3 mg/kg dose had a significant protective effect (Fig. 1, p < 0.0005 LogRank Mantel-Cox). *Scn1a* ^+/−^ mice pre-treated with 3 mg/kg SGE-516 had an elevated temperature threshold for GTCS of 42.5 ± 0.02 °C and 38% (5 of 13) mice were seizure-free, while 94% (15 of 16) of vehicle-treated control mice seized with a threshold temperature of 42.1 ± 0.09 °C. Plasma was collected at the conclusion of the hyperthermia protocol and SGE-516 levels were measured. The 3 mg/kg SGE-516 dose resulted in plasma exposure levels comparable to those previously identified as anticonvulsant in acute seizure models (~80 ng/mL), while the 0.1 and 1 mg/kg doses resulted in plasma exposure levels below the anticonvulsant range (Table 1, Supplementary Figure S1)^12^.

**Table 1:** Plasma exposure levels in mice following administration of SGE-516.

Experiment	Dose (length of treatment)^*^	Route of Administration	^†^Plasma Exposure (ng/mL) ^‡^	# of samples
Hyperthermia Threshold	0.1 mg/kg (1 hour)	IP	BQL	n/a
	1 mg/kg (1 hour)	IP	54.7 ± 13.6	6
	3 mg/kg (1 hour)	IP	114 ± 21.0	6
Sub-chronic oral administration	8 mg/kg/day (24 hours)	Oral (Chow)	BQL	6
	8 mg/kg/day (72 hours)	Oral (Chow)	9 ± 1.72	6
	40 mg/kg/day (24 hours)	Oral (Chow)	29 ± 13.3	5
	40 mg/kg/day (48 hours)	Oral (Chow)	9 ± 1.72	5
	40 mg/kg/day (72 hours)	Oral (Chow)	28 ± 5.09	5
	120 mg/kg/day (24 hours)	Oral (Chow)	84 ± 22.3	5
	120 mg/kg/day (48 hours)	Oral (Chow)	51 ± 19.0	5
	120 mg/kg/day (72 hours)	Oral (Chow)	64 ± 18.8	5
	120 mg/kg/day (30 days)	Oral (Chow)	53 ± 14.8	10
	267 mg/kg/day (24 hours)	Oral (Chow)	239.8 ± 75.3	5
	267 mg/kg/day (48 hours	Oral (Chow)	113.6 ± 38.6	5
	267 mg/kg/day (72 hours)	Oral (Chow)	182.6 ± 50.5	5
Spontaneous seizure monitoring	120 mg/kg/day (12 days)	Oral (via chow)	37.3 ± 7.0	12

^*^Length of treatment indicates the time interval between treatment initiation and plasma sample collection.

^†^Minimal effective concentration of SGE-516 for anticonvulsant activity is between 35 and 84 ng/mL^12^. ^**‡**^Concentrations represent average ± SEM per dose. BQL: below quantification limit.

### Effect of SGE-516 treatment on survival of *Scn1a*^*+/−*^ Dravet mice

**Figure 2:**
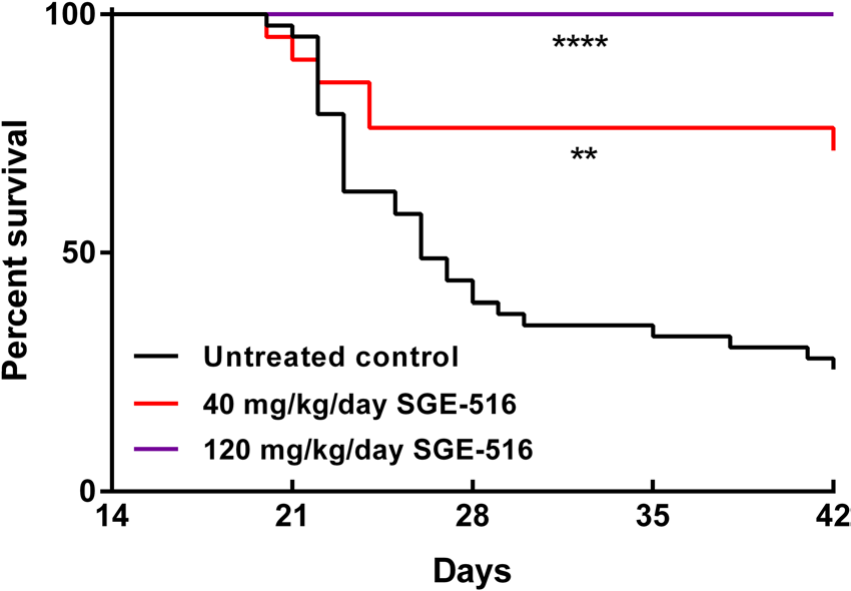
Effect of chronic oral administration of SGE-516 on survival of *Scn1a* ^*+/−*^ mice. Kaplan Meier survival curves comparing *Scn1a* ^+/−^ mice maintained on control chow, 40 mg/kg/day or 120 mg/kg/day of SGE-516. Treatment with SGE-516 or control chow commenced at P18 and survival was assessed until P42. The 40 mg/kg/day SGE-516 dose significantly improved survival of *Scn1a* ^+/−^ mice (**p < 0.002), while the 120 mg/kg/day SGE-516 dose completely prevented premature lethality in *Scn1a* ^+/−^ mice (****p < 0.0001). P-values were determined by LogRank Mantel-Cox (*n* = 43 for control; n = 21 for 40 mg/kg/day; n = 16 for 120 mg/kg/day).

*Scn1a* ^+/−^ mice have a significantly reduced lifespan, with approximately 50% lethality by one month of age^15,16^. We evaluated the effect of chronic SGE-516 treatment on *Scn1a* ^+/−^ survival. SGE-516 was administered orally by providing *ad libitum* access to mouse chow formulated with the compound. Pilot pharmacokinetic experiments (see methods) established that doses in chow of 40 mg/kg/day or 120 mg/kg/day resulted in plasma levels within the range shown to be anticonvulsant in mice (Table 1, Supplementary Figure S1)^12^. Treatment with SGE-516 chow at 40 mg/kg/day or 120 mg/kg/day was initiated on postnatal day 18 (P18) and survival was monitored until 6 weeks of age. Both SGE-516 doses significantly improved survival of *Scn1a* ^+/−^ mice (Fig. 2). Relative to untreated controls that had 25% survival to 6 weeks of age, treatment with 40 mg/kg/day of SGE-516 significantly improved survival (p < 0.002 LogRank Mantel-Cox), with 71% of *Scn1a* ^+/−^ mice surviving to 6 weeks. Treatment with 120 mg/kg/day of SGE-516 completely prevented premature lethality in *Scn1a* ^+/−^ mice (p < 0.0001 LogRank Mantel-Cox). A separate cohort of mice was maintained on 120 mg/kg/day SGE-516 for 30 days to determine exposure levels and monitor for potential adverse effects with chronic treatment. Overt/profound sedation or weight loss was not observed and plasma exposure levels were similar to the 72 hour time point (Table 1, Supplementary Figure S1). Based on the robust protective benefit on survival and the absence of overt adverse effects, we used the 120 mg/kg/day SGE-516 dose for the remaining experiments.

### Effect of SGE-516 treatment on spontaneous seizures in *Scn1a*^*+/−*^ Dravet mice

**Figure 3:**
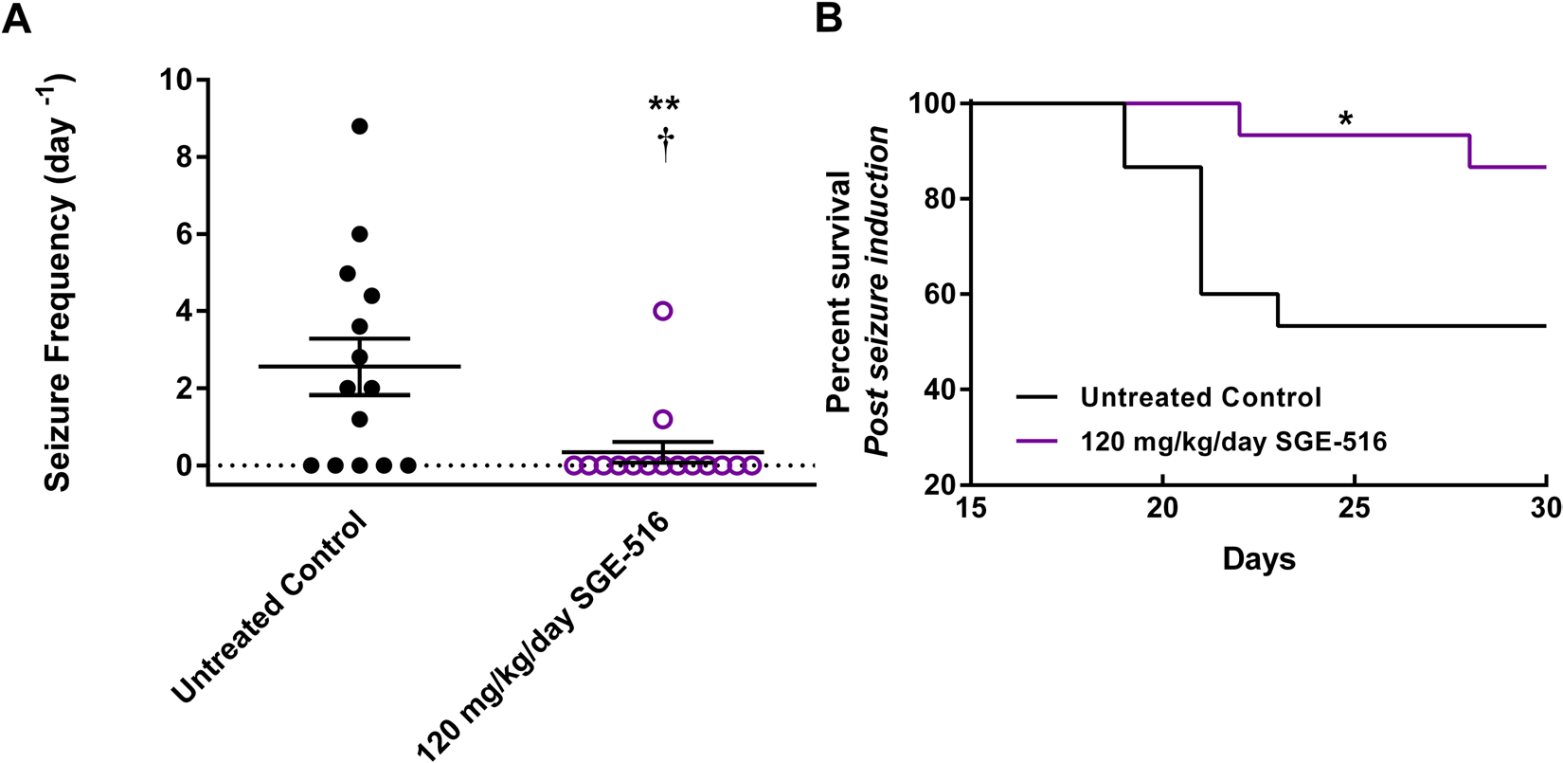
Effect of SGE-516 treatment on spontaneous seizure frequency and survival following a hyperthermia-induced seizure in *Scn1a* ^*+/−*^ Dravet mice. GTCS frequency and survival of individual *Scn1a* ^+/−^ mice maintained on control or 120 mg/kg/day SGE-516 chow are shown. Treatment with SGE-516 was initiated at P18 immediately following induction of a single hyperthermia-priming seizure. (**A**) Spontaneous GTCS frequency was quantified over a 60-hour video recording period starting at 12:00 on P19 through 24:00 on P21. Treatment with 120 mg/kg/day SGE-516 significantly increased the percentage of *Scn1a* ^+/−^ mice that were seizure-free (**p < 0.008, Fisher’s exact) and reduced average daily seizure frequency (0.35 ± 0.27) compared to untreated control *Scn1a* ^+/−^ mice (2.56 ± 0.73) (^†^p < 0.005, Mann-Whitney). Average seizure frequency is depicted by the thick horizontal line and error bars represent standard error of the mean (SEM), with *n* = 14–15 mice per treatment group. (**B**) Thirty-day survival was assessed following a hyperthermia-induced priming seizure and spontaneous seizure monitoring. Treatment with 120 mg/kg/day of SGE-516 significantly improved survival of *Scn1a* ^+/−^ mice compared to untreated *Scn1a* ^+/−^ mice (*p < 0.04). P value was determined by LogRank Mantel-Cox with *n* = 15 per treatment group.

We recently developed a novel paradigm in which we induced a single, brief GTCS with hyperthermia on P18 and subsequently assessed spontaneous GTCS frequency thereafter^14^. This hyperthermia-priming protocol increased the occurrence of spontaneous seizures for several days compared to naïve *Scn1a* ^+/−^ mice, improving discrimination power to identify a protective effect of treatment. To evaluate SGE-516 in this paradigm, on P18 we induced a single hyperthermia-priming seizure in *Scn1a* ^+/−^ mice, rapidly terminated the seizure by cooling and then commenced sub-chronic treatment with control or 120 mg/kg/day SGE-516 chow. *Scn1a* ^+/−^ mice were then continuously video recorded for 60 hours between 12:00 on P19 and 24:00 on P21 and spontaneous GTCS frequency was assessed from video-records. Treatment with 120 mg/kg/day of SGE-516 chow significantly reduced spontaneous GTCS frequency compared to untreated controls (Fig. 3A). SGE-516 treated *Scn1a* ^+/−^ mice averaged 0.35 ± 0.27 seizures per day, a significant reduction compared to untreated *Scn1a* ^+/−^ controls which experienced an average of 2.56 ± 0.73 seizures per day (p < 0.005). Furthermore, only 13% (2 of 15) of 120 mg/kg/day SGE-516 treated *Scn1a* ^+/−^ mice exhibited seizures during the observation period, while 64% (9 of 14) of untreated control *Scn1a* ^+/−^ mice experienced GTCS (p < 0.0078). Following cessation of video monitoring, SGE-516 treatment was continued and survival was monitored to P30. Treatment with SGE-516 significantly improved survival, with 87% of *Scn1a* ^+/−^ mice on SGE-516 surviving to P30 compared to only 53% of controls (Fig. 3B, p < 0.04). At the conclusion of the survival study on P30, plasma was collected and SGE-516 levels were measured to confirm exposures were in the previously reported therapeutic range (Table 1, Supplementary Figure S1)^12^.

### Effect of SGE-516 treatment on electrographic seizures in *Scn1a*^*+/−*^ Dravet mice

**Figure 4:**
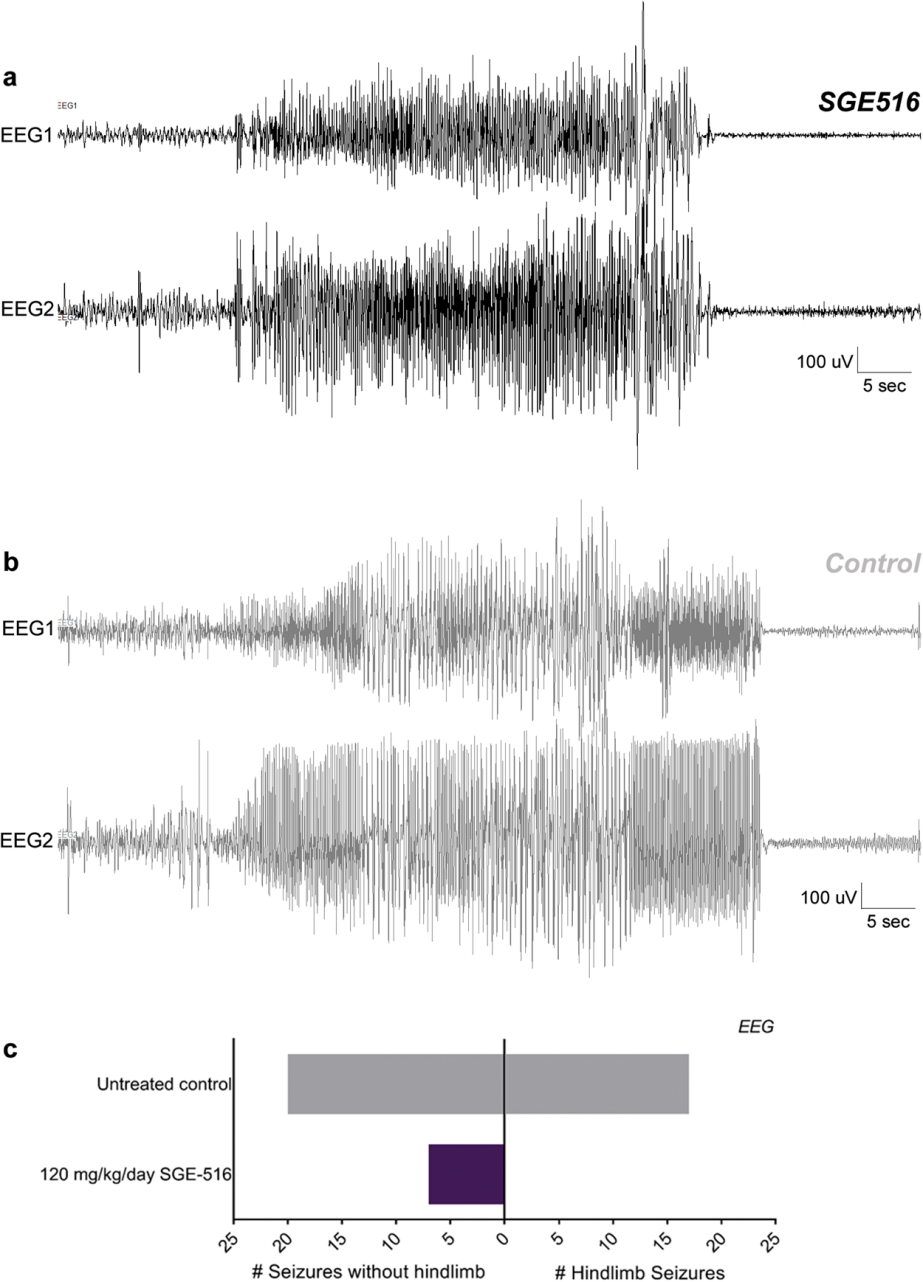
Effect of SGE-516 treatment on electrographic seizures Representative EEGs during an ictal event from *Scn1a* ^+/−^ mice maintained on control or 120 mg/kg/day SGE-516 chow. *Scn1a* ^+/−^ mice were monitored by video-EEG to evaluate electrographic ictal events and their behavioral correlates. EEG1 represents recordings between right posterior and left posterior electrodes. EEG2 represents recordings between right anterior to left posterior electrodes. (**A**) Typical EEG trace from a rarely occurring GTCS event in an *Scn1a* ^+/−^ mouse treated with SGE-516 at 120 mg/kg/day. Coincident electrographic and behavioral components were observed in the EEG and video records, respectively. All electrographic seizures in SGE-516-treated mice coincided with an observable generalized tonic-clonic convulsion. EEG traces from ictal events in SGE-516 treated mice were indistinguishable from GTCS events observed in untreated controls. (**B**) Typical EEG trace from a GTCS event in untreated *Scn1a* ^+/−^ control mice. Coincident electrographic and behavioral components were identified in the EEG and video records, respectively. (**C**) The total number of spontaneous GTCS with or without full tonic hindlimb extension of untreated control (grey bar) and 120 mg/kg/day SGE-516 (purple bar) treated mice is displayed.

Video-electroencephalography (EEG) is the gold standard for validating the ability of anti-seizure compounds to reduce or prevent spontaneous electrographic seizures. Furthermore, EEG can distinguish between compounds which only prevent the motor component from those that abolish both behavioral and electrographic seizures. This is complementary to video-only analysis, which has higher throughput but relies on observation of behavioral seizures. We performed video-EEG monitoring to determine whether SGE-516 prevented electrographic seizure activity or if it only suppressed the behavioral component of GTCS events. At P18, *Scn1a* ^+/−^ mice were provided *ad libitum* access to control or 120 mg/kg/day SGE-516 chow. On P19-20, mice were implanted with prefabricated EEG headmounts and allowed to recover for 48 hours. Beginning on P21-22, video-EEG data was continuously collected though P26-27. *Scn1a* ^+/−^ mice treated with 120 mg/kg/day SGE-516 were video-EEG monitored for a total of 840 hours (average 93.3 ± 8.4 hours per mouse; range 48-120) and control mice were monitored for a total of 1467 hours (average 91.7 ± 6.8 hours per mouse; range 22-120). *Scn1a* ^+/−^ mice on 120 mg/kg/day SGE-516 treatment had an average seizure frequency of 0.16 ± 0.12 seizures per day, while untreated controls had an average seizure frequency of 1.2 ± 0.79 seizures per day (Table 2). However, the percentage of *Scn1a* ^+/−^ mice that were seizure-free was high in both groups (81% untreated, 78% SGE-516), precluding statistical comparison of seizure frequency in this cohort. This was not entirely unexpected, as we previously observed that surgery and/or post-surgical supportive care reduces seizure burden and improves survival of *Scn1a* ^+/−^ mice^14,17^. Despite this limitation, when rare electrographic seizures occurred in SGE-516 treated mice, the electrographic events coincided with observable generalized tonic-clonic convulsions in the video. Electrographic and behavioral events in SGE-516-treated mice were indistinguishable from those observed in untreated *Scn1a* ^+/−^ mice (Fig. 4A,B). This suggests that SGE-516 treatment does not simply obscure the behavioral component of GTCS events.

**Table 2:** Video-EEG analysis of mice treated with SGE-516.

**Group**	**Monitoring Age Range**	**# of mice**	**Total hours**	**Average hours per mouse (range)**	**Total Number of GTCS**	**Average GTCS frequency (day** ^**−1**^ )*	**% of GTCS with Tonic Hindlimb Extension**	**% of mice GTCS-free**
SGE-516	P22-P27	9	840	93.3 ± 8.4 (48–120)	7	0.16 ± 0.12	0	78
Untreated	P21-P26	16	1467	91.7 ± 6.8 (22–120)	38	1.20 ± 0.79	47	81

GTCS: generalized tonic-clonic seizure; *calculated by averaging individual seizure frequencies for each treatment group.

In previous studies, we observed that sub-chronic treatment with some anticonvulsants modified GTCS severity in *Scn1a* ^+/−^ mice by attenuating advancement to the most severe stage of tonic hindlimb extension^14^. Therefore, we also evaluated the effect of SGE-516 on GTCS severity. All seizures observed from the video-EEG records included generalized tonic-clonic activity with loss of posture, while a subset advanced to the more severe stage of tonic hindlimb extension. All GTCS events from the video-EEG records were scored for advancement to the most severe stage of full tonic hindlimb extension (hindlimbs at a 180° angle to the torso). Of the 7 GTCS observed in 120 mg/kg/day SGE-516 treated mice, none progressed to full tonic hindlimb extension (Fig. 4C, Table 2). In contrast, in the untreated control group, 47%, (18 of 38) of the GTCS advanced to tonic hindlimb extension, consistent with previous reports (Fig. 4C, Table 2)^14^.

## Discussion

SGE-516 treatment provided protection against hyperthermia-induced seizures, reduced spontaneous seizure frequency, and prolonged survival in *Scn1a* ^+/−^ mice. This work provides the first evidence of SGE-516 activity in a mouse model of Dravet syndrome. Furthermore, it supports the development of synthetic neuroactive steroids as potential anticonvulsant therapies for refractory genetic epilepsies.

Previous work showed that the endogenous neuroactive steroid allopregnanolone, a potent PAM of GABA_A_ receptors, has anticonvulsant activity in several rodent models, including PTZ seizure induction, 6 Hz threshold testing and status epilepticus induced by pilocarpine or kaniate^9,10,18^. Studies with SGE-516 have also demonstrated protection against induced seizures in the PTZ, 6 Hz, corneal kindling and organophosphate status epilepticus models^12,13^. Together, these data suggest that neuroactive steroids, such as SGE-516, warrant further investigation as potential novel treatments for a wide range of seizure disorders.

While roughly 20 anticonvulsants are currently FDA approved for the treatment of epilepsy, first-line therapy fails in approximately one-third of children^19–21^. Dravet syndrome is generally refractory to currently available AEDs. Polytherapy with valproic acid, clobazam and stiripentol has shown some success in reducing seizure severity and frequency for Dravet syndrome patients, although seizure-freedom is rare^22,23^. Recently, we assessed each of these drugs individually and in combination in the *Scn1a* ^+/−^ mouse model using similar assays to the current study^14^. Valproic acid was protective against hyperthermia-induced seizures at supratherapeutic doses, while stiripentol was not effective in any of these assays^14^. *Scn1a* ^+/−^ mice treated with clobazam at doses corresponding to the human therapeutic range had an increased temperature threshold for hyperthermia-induced seizures and reduced spontaneous seizure frequency compared to controls^14,24^. However, clobazam had no effect on long-term survival of *Scn1a* ^+/−^ Dravet mice, even at supratherapeutic doses^14,24^. In contrast, SGE-516 was protective in all three assays, including survival. This suggests that the mechanisms or pathways underlying the seizure and premature sudden death phenotypes of *Scn1a* ^+/−^ Dravet mice are separable and may be differentially modulated by benzodiazepines versus neuroactive steroids like SGE-516. Future studies addressing the effect of SGE-516 on central control of the cardiovascular and respiratory systems, as well as effects in the periphery, will be necessary to understand how SGE-516 protects *Scn1a* ^+/−^ mice from premature death.

Together, the results of the current study demonstrating protective benefit of SGE-516 in a Dravet mouse model and previous reports of protective effects in traditional anticonvulsant screening models support further investigation of neuroactive steroids as potential novel anticonvulsant compounds in the treatment of various refractory epilepsies.

## Methods

### Mice

*Scn1a* ^*tm1Kea*^ mice, with deletion of the first coding exon, were generated by homologous recombination in TL1 ES cells (129S6/SvEvTac) and genotyped as previously described^15^. The *Scn1a* ^+/−^ line is maintained by continuous backcrossing to 129S6/SvEvTac (129) (Taconic Biosciences, Hudson, NY, USA). Mice for experiments were generated by crossing 129.*Scn1a* ^+/−^ mice with C57BL/6 J (B6) (#000664, Jackson Laboratory, Bar Harbor, ME, USA), resulting in [129 x B6]F1.*Scn1a* ^+/−^ offspring, referred to herein as *Scn1a* ^+/−^ mice. Mice were maintained in a Specific Pathogen Free (SPF) barrier facility with a 14-hour light/10-hour dark cycle and access to food and water *ad libitum*. Both female and male *Scn1a* ^+/−^ mice were utilized for all experiments. There were no significant differences between sexes on any measurements, so groups were collapsed across sex. All animal care and experimental procedures were approved by the Northwestern University Animal Care and Use Committee in accordance with the National Institutes of Health Guide for the Care and Use of Laboratory Animals. The principles outlined in the ARRIVE (Animal Research: Reporting of *in vivo* Experiments) guideline and Basel declaration (including the 3 R concept) were considered when planning experiments.

### Drug formulation and dosing

For acute administration, SGE-516 was solubilized in 2% 2-Hydroxypropyl-β-cyclodextrin (HPBCD) (Acros Organics, New Jersey, USA) by sonication until a clear solution was obtained and was administered by intraperitoneal (IP) injection. Previous work from our laboratory demonstrated that up to 5% HPBCD was an inert vehicle in the hyperthermia-induced seizure assay^14^. For chronic administration, chow pellets were custom formulated using Teklad 7912 (Envigo, Indianapolis, Indiana, USA) chow base with addition of SGE-516 powder at 30 mg per kg, 150 mg per kg, 450 mg per kg or 1000 mg per kg of chow (Research Diets, New Brunswick, New Jersey, USA). The estimated daily doses were 8 mg/kg/day, 40 mg/kg/day, 120 mg/kg/day or 267 mg/kg/day respectively, based on an assumed consumption of 4 grams of chow per day and average body weight of 15 grams (http://www.researchdiets.com/resource-center/typical-food-intake). Control chow was Teklad 7912 without compound (Envigo). Mice maintained on SGE-516 or control diet were monitored for general health daily and body weight was measured every other day. No signs of overt/profound sedation or weight loss were observed in mice on control or SGE-516 chow.

### Dose-finding for chow formulation

A pilot pharmacokinetic study was performed to identify two doses of SGE-516 chow that produced plasma exposure levels comparable to those that were previously shown to be anticonvulsant in mice^12^. At postnatal day 18 (P18), mice initiated treatment with either 8, 40, 120 or 267 mg/kg/day SGE-516 chow and plasma was collected at 24, 48 and 72 hours to assay SGE-516 levels. The 8 mg/kg/day SGE-516 dose resulted in plasma levels below the quantification limit, while the 267 mg/kg/day dose resulted in plasma levels significantly above the target range (Table 1, Supplementary Figure S1). The 40 mg/kg/day and 120 mg/kg/day SGE-516 chows produced plasma exposures within the target range and were used for chronic dosing studies (Table 1, Supplementary Figure S1).

### Hyperthermia-induced seizures

Hyperthermia-induced seizure assays were performed on *Scn1a* ^+/−^ mice at P19-20. Core body temperature was monitored with a RET-3 rectal temperature probe (Physitemp Instruments, Inc, New Jersey, USA) and controlled by a heat lamp connected to a rodent temperature regulator (TCAT-2DF, Physitemp) reconfigured with a Partlow 1160 + controller (West Control Solutions, Brighton, UK). SGE-516 was administered by IP injection and mice were returned to their home cage for 40 minutes. The temperature probe was inserted at 40 minutes post-injection and mice acclimated to probe and test environment for 5 minutes. At 45 minutes post-injection, body temperature was raised 0.5 °C every two minutes until the onset of the first clonic convulsion with loss of posture or until 42.5 °C was maintained for 5 minutes. Mice that did not experience a GTCS during the 5-minute hold at 42.5 °C were considered seizure-free. Threshold temperatures were compared using the time-to-event analysis with P values determined with LogRank Mantel-Cox test. P values < 0.05 were considered statistically significant.

### Determination of SGE-516 plasma concentrations

After experiments, SGE-516 treated mice were anesthetized with isoflurane and whole blood was collected by cardiac puncture. Plasma was isolated by centrifugation in K_2_EDTA tubes (2000 x g for 10 minutes, 4 °C). All samples were stored at -80 °C until assayed. Plasma SGE-516 concentrations were assayed by LC-MS/MS analysis as previously described^12^ (PharmaCadence Analytical Services, Hatfield, PA, USA).

### Six week survival monitoring

At P18, *Scn1a* ^+/−^ mice were weaned and block randomized into standard vivarium holding cages with *ad libitum* access to control, 40 mg/kg/day SGE-516 or 120 mg/kg/day SGE-516 chow. Each cage contained 4–5 mice of the same age and sex, with wild-type littermates included as needed. Survival was monitored until 6 weeks of age. During that time, all mice were monitored daily for general health and any mouse visibly unhealthy (e.g. underweight, dehydrated, poorly groomed, or immobile) was excluded from the study. No signs of overt/profound sedation or weight loss were noted with SGE-516 treatment. All recorded deaths were sudden and unexpected, occurring in otherwise healthy appearing animals. Survival statistics were calculated using time-to-event analysis with LogRank Mantel-Cox test. P values < 0.05 were considered statistically significant.

### Spontaneous seizure monitoring

Spontaneous GTCS frequency was assessed following hyperthermia-priming of *Scn1a* ^+/−^ mice with a single, brief hyperthermia-induced seizure at P18 as previously described^14^. Briefly, at P18, *Scn1a* ^+/−^ mice were subjected to the above described hyperthermia-induced seizure protocol. After GTCS onset, mice were rapidly cooled to 37 °C. Mice were then placed in a monitoring cage with *ad libitum* access to control or 120 mg/kg/day SGE-516 chow. Spontaneous seizure activity was captured by continuous video monitoring as previously described^24^. Seizures were counted offline beginning at 12:00 on P19 through 24:00 on P21 (60 hours). Seizure frequency was determined for each subject by dividing the total number of seizures by 2.5 days. If a mouse did not complete 60 hours of monitoring (spontaneous death), the total number of seizures was divided by total hours monitored and converted to seizure frequency per day. Following cessation of video recording, mice continued on control or SGE-516 chow and survival was monitored until P30. Seizure frequencies were compared between treatment groups with a Mann-Whitney U-test and survival was compared using logrank Mantel-Cox test. P values < 0.05 were considered statistically significant.

### Video-EEG Monitoring

At P18, *Scn1a* ^+/−^ mice were provided *ad libitum* access to control or 120/mg/kg/day SGE-516 chow. For this experiment, *Scn1a* ^+/−^ mice were not primed with a hyperthermia-induced GTCS. At P19-20, SGE-516-treated mice and control mice were implanted with prefabricated EEG headmounts (Pinnacle Technology, Kansas, USA). Briefly, mice were anesthetized with isoflurane and placed in a stereotaxic frame. Headmounts with four stainless steel screws that served as cortical surface electrodes were affixed to the skull with dental acrylic. Anterior screw electrodes were 0.5–1 mm anterior to bregma and 1 mm lateral from the midline. Posterior screws were 4.5–5 mm posterior to bregma and 1 mm lateral from the midline. EEG1 represents recordings from right posterior to left posterior (interelectrode distance ~2 mm). EEG2 represents recordings from right anterior to left posterior (interelectrode distance ~5 mm). The left anterior screw served as the ground connection.

Following 48 hours of recovery, tethered EEG and video data were continuously collected from freely moving mice for 5 days, as previously described^24^. *Scn1a* ^+/−^ mice on SGE-516 chow (n = 9) were monitored for a total of 840 hours. *Scn1a* ^+/−^ mice on control chow (n = 16) were monitored for a total of 1467 hours (Table 2). Additional mice were included in the control chow group to compensate for anticipated premature deaths. EEG was acquired with Sirenia acquisition software (Pinnacle Technology). Video-EEG records were scored manually offline by a reviewer blinded to treatment. Individual seizure frequencies were determined for each subject and then averaged to obtain an average seizure frequency for each treatment group. However, baseline seizure frequency in the untreated control group was low. Therefore, data from this experiment were not used for statistical comparisons of seizure frequency, but instead were used for qualitative comparison of electrographic events.

### Data availability

The datasets generated and analyzed during the current study are available from the corresponding author on reasonable request.

## Electronic supplementary material

Supplemental Figure S1
