# Supplementary material for: The synthetic neuroactive steroid SGE-516 reduces seizure burden and improves survival in a Dravet syndrome mouse model
Source: Sci Rep. 2017 Nov 10;7:15327. doi: 10.1038/s41598-017-15609-w (PMC5681541; doi:10.1038/s41598-017-15609-w)
Supplement: Supplementary file 1 — Supplemental Figure S1 [file 41598_2017_15609_MOESM1_ESM.pdf]

Hawkins NA, Lewis M, Hammond RS, Doherty JJ, Kearney JA. The synthetic neuroactive steroid SGE-516 reduces seizure burden and improves survival in a Dravet syndrome mouse model.

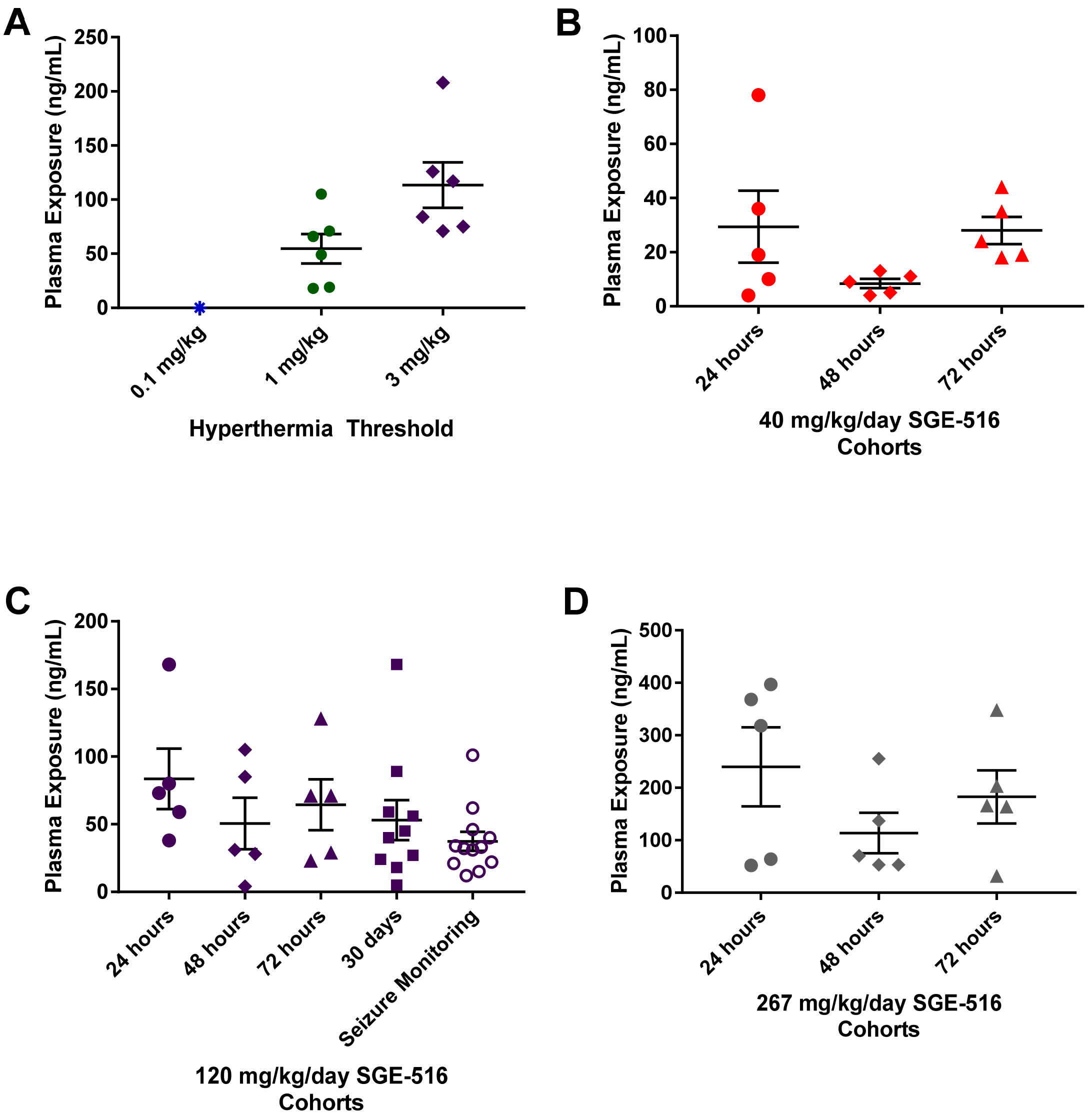

Supplementary Figure S1. Individual plasma exposures from experiments.
A. Plasma levels from *Scn1a*$^{+/-}$ mice that received an acute IP dose of SGE-516 and underwent hyperthermia-threshold seizure testing. Plasma samples were collected immediately following the seizure induction protocol. The 0.1 mg/kg SGE-516 dose was BQL (blue asterisk). Average is depicted by the horizontal line and error bars are SEM, n=6/group. **B**. Plasma levels from *Scn1a*$^{+/-}$ mice maintained on 40 mg/kg/day SGE-516 orally via chow. Plasma samples were collected at a variety of times (24, 48 and 72 hours) to verify plasma exposures. Average is depicted by the horizontal line and error bars are SEM, n=5/group. **C**. Plasma levels from *Scn1a*$^{+/-}$ mice maintained on 120 mg/kg/day SGE-516 orally via chow. Plasma samples were collected at a variety of times (24, 48 and 72 hours, 30 days) and also include *Scn1a*$^{+/-}$ mice that were monitored for spontaneous seizure frequency to verify plasma exposures following 12 days of treatment. Average is depicted by the horizontal line and error bars are SEM, n=5-12/group. **D**. Plasma levels from *Scn1a*$^{+/-}$ mice maintained on 267 mg/kg/day SGE-516 orally via chow. Plasma samples were collected at a variety of times (24, 48 and 72 hours) to verify plasma exposures. Average is depicted by the horizontal line and error bars are SEM, n=5/group.
